# Mosquitocidal and Anti-Inflammatory Properties of The Essential Oils Obtained from Monoecious, Male, and Female Inflorescences of Hemp (*Cannabis sativa* L.) and Their Encapsulation in Nanoemulsions

**DOI:** 10.3390/molecules25153451

**Published:** 2020-07-29

**Authors:** Paolo Rossi, Alessia Cappelli, Oliviero Marinelli, Matteo Valzano, Lucia Pavoni, Giulia Bonacucina, Riccardo Petrelli, Pierluigi Pompei, Eugenia Mazzara, Irene Ricci, Filippo Maggi, Massimo Nabissi

**Affiliations:** 1 School of Biosciences and Veterinary Medicine, University of Camerino, 62032 Camerino, Italy; paolo.rossi@unicam.it (P.R.); alessia.cappelli@unicam.it (A.C.); matteo.valzano@unicam.it (M.V.); irene.ricci@unicam.it (I.R.); 2 School of Pharmacy, University of Camerino, 62032 Camerino, Italy; oliviero.marinelli@unicam.it (O.M.); lucia.pavoni@unicam.it (L.P.); giulia.bonacucina@unicam.it (G.B.); riccardo.petrelli@unicam.it (R.P.); pete.pompei@unicam.it (P.P.); eugenia.mazzara@unicam.it (E.M.)

**Keywords:** malaria, hemp, *Anopheles gambiae*, *Anopheles stephensi*, green pesticides, anti-inflammatory

## Abstract

Among the various innovative products obtainable from hemp (*Cannabis sativa* L.) waste biomass originating from different industrial processes, the essential oil (EO) deserves special attention in order to understand its possible application in different fields, such as cosmetics, pharmaceuticals, and botanical insecticides. For the purpose, in the present work, we studied the chemical composition of EOs obtained from different hemp varieties, namely Felina 32 and Carmagnola Selezionata (CS) using monoecious, male, and female inflorescences, and we evaluated their mosquitocidal activities on larvae and pupae of two main malaria vectors, *Anopheles gambiae* and *An. stephensi*. Then, in order to evaluate the safe use of hemp EOs for operators, the potential pro- or anti-inflammatory effect of hemp EOs together with their toxicological profile were determined on dermal fibroblasts and keratinocytes. Given the promising results obtained by insecticidal and anti-inflammatory studies, a preliminary evaluation of EOs encapsulation into nanoemulsions (NEs) has been performed with the aim to develop a formulation able to improve their poor physicochemical stability. Felina 32 and CS inflorescences provided EOs with an interesting chemical profile, with monoterpene and sesquiterpene hydrocarbons as the major components. This study highlighted the potential application of male inflorescences, which are usually discharged during hemp product processing. These EOs could be exploited as potential sustainable and eco-friendly insecticides, given their capability to be toxic against mosquitoes and the possibility to use them to prepare stable and safe formulations. The LC_50_ values found in this study (<80 ppm) are lower, on average, than those of many plant EOs, with the advantage of using an industrial waste product. From MTT assay and gene and protein expression analysis, EOs showed no cytotoxicity at the appropriate doses and exerted an anti-inflammatory effect on the human cell lines tested. These findings encourage further applied research on hemp EOs in order support their industrial exploitation.

## 1. Introduction

The cultivation of hemp (*Cannabis sativa* L.) in the European Union is steadily increasing, thanks to the community policies aimed at improving low-impact agricultural techniques and at valorizing the agricultural and quality productions. Notably, in Italy, around 4000 ha are cultivated with hemp by farms, for an estimated income of about 40 million euros. In this scenario, it is very important to develop innovative products from this multipurpose crop. In this regard, the hemp essential oil (EO), which is a mixture of volatile compounds produced in the bract glandular hairs of the plant inflorescences, is a niche product that is gaining interest by companies for its possible application in different fields, such as organic agriculture, pharmaceuticals, cosmetics, and botanical insecticides [1]. Hemp EO is mostly made up by monoterpenes and sesquiterpenes, with a lower contribution of the cannabinoid fraction [2,3]. Overall, plant EOs have shown potential to be used for their antimicrobial, anti-inflammatory, insecticidal, and parasiticidal properties [4,5,6,7,8]. In this respect, hemp EO has hitherto received little attention.

Malaria causes 219 million cases and 435,000 related deaths each year [9]. Female Anopheline mosquitoes inject *Plasmodium* parasites into the human circulatory system during the blood meal [10]. *Anopheles gambiae* and *An. stephensi* are the main malaria vectors in Africa and Asia, respectively [11].

Malaria control strategies are based on vector control through insecticide-treated mosquito nets and house spraying by indoor residual spraying. In endemic areas, synthetic pesticides, such as pyrethroids, have been used for larval and adult mosquito control for decades [12]. Besides the high costs of applications, their excessive use is causing development of insecticide resistance [13,14]. 

The need for sustainability of malaria control strategies and growing insecticide-resistance are inducing the exploration of new natural pesticides. In this context, plant EOs and their bioactive molecules can constitute a potential weapon against insect vectors and are considered as effective larvicides with fewer mosquito resistance issues [15,16]. Moreover, they are biodegradable and low-cost products that could be used in malaria control programs [17]. Among various crops available as industrial sources of EO, hemp, *Cannabis sativa* L. (Cannabaceae), has recently received growing interest for its potential use as a botanical insecticide. Indeed, the plant inflorescences have developed as a natural weapon against phytophagous insects, secreting essential secondary metabolites such as cannabinoids and volatile terpenes [18,19]. In previous reports, *C. sativa* EO proved to be effective against aphids, houseflies, and ticks [2,3,20]. Moreover, this EO proved to be eco-friendly since no toxicity was detected on non-target organisms such as earthworms and ladybirds [3]. Notably, the possible development of hemp EO-based insecticides would enjoy the following advantages: (i) lack of similar products on the market; (ii) low cost of raw material (frequently as a byproduct); (iii) availability of agricultural land for plant cultivation over the world; (iv) increasing demand for eco-friendly and safe products; (v) possibility to split the end products into other fields, such as cosmetics and pharmaceuticals [1]. 

The preclinical cell model for assessing safety and toxicity of the chemical and plant-derived topical insect repellents and insecticides have been applied using human keratinocytes and dermal fibroblasts. The skin responds to a variety of physical stimuli through modulation of specific pathways. The epidermis is mainly constituted of keratinocytes, which represent 95% of the epidermal cells and are involved in the initiation and perpetuation of skin inflammatory and immunological responses [21,22]. Moreover, fibroblasts strictly interact with keratinocytes and are involved in wound healing [23]. Keratinocytes, as well as fibroblasts, release several proinflammatory mediators, including interleukin-1 beta (IL-1 beta), IL-6, IL-8, tumor necrosis factor-alpha (TNF alpha), under inflammation stimuli, and several studies have indicated that etoposide (ETO) triggers cytokine production in human cell lines in vitro [24,25]. 

On this basis, the main objective of this study was to verify if the EOs obtained from inflorescences of hemp may be of interest for the manufacturing of botanical insecticides, as well as their safety profile for operators. For the purpose, we selected two different hemp varieties, namely, Felina 32 and Carmagnola CS, and distilled the EOs from fresh and dry material and from monoecious, male, and female inflorescences using two different extraction techniques—steam distillation (SD) and hydrodistillation (HD)—in order to verify whether the variability of the chemical profiles may impact on the biological activities. The potential toxic effects of the selected hemp EOs were assessed on larvae and pupae of *An. stephensi* and *An. gambiae* mosquitoes. Secondly, we investigated the potential pro- or anti-inflammatory effect of hemp EOs, in order to evaluate their safe use for operators, by analyzing the proinflammatory cytokines release in ETO-treated human keratinocytes and fibroblasts. Finally, in order to reach a real-world application of hemp EOs, a preliminary evaluation of their encapsulation into nanoemulsions (NEs) has been performed. Nanoencapsulation helps to overcome the poor physicochemical stability, limited water solubility, high volatility, and thermal decomposition that usually characterize EOs [26]. Thus, the possibility to encapsulate EOs and plant extracts inside NEs ensures stability and protection of components, an increase of biological activity, and an improved solubility. For these reasons, the hemp EO NEs have been successfully developed, characterized, and their stability over time evaluated.

## 2. Results

### 2.1. Chemical Composition of C. sativa Essential Oils

The three EOs from the monoecious Felina 32 and female and male inflorescences of CS presented a yield of 0.10, 0.15, and 0.07%, respectively. These values were consistent with those previously reported by different authors [1,3,27]. Table 1 shows the chemical profiles of the EOs, with a total of 31 identified compounds accounting for 97.6–99.9% of the overall compositions.

**Figure 1:**
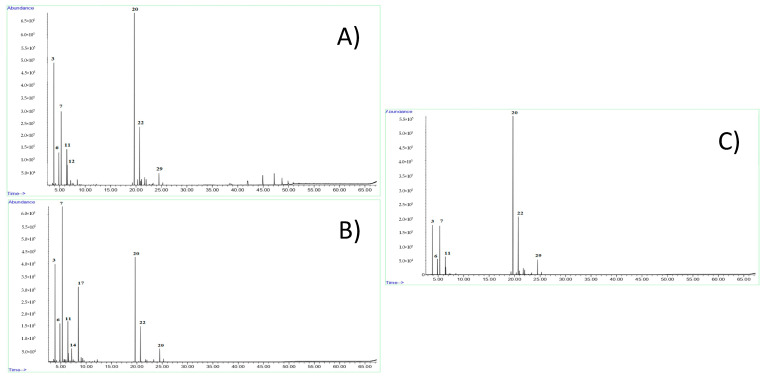
GC-MS chromatograms of the hemp essential oils obtained from the monoecious Felina 32 inflorescence (**A**), and female (**B**) and male (**C**) inflorescences of CS. Peak numbering refers to Table 1.

The major fraction of the EOs from Felina 32 and male CS was represented by sesquiterpene hydrocarbons (52.1% and 67.7%, respectively), with (*E*)-caryophyllene (34.8% and 47.2%, respectively) and α-humulene (11.4% and 15.1%, respectively) as the major compounds (Figure 1). The monoterpene hydrocarbons constituted the second major group in these EOs (40.6% and 26.6%, respectively), with α-pinene (15.1% and 8.0%, respectively) and myrcene (11.8% and 10.6%, respectively) as the most abundant compounds.

On the other hand, the EO obtained from the female CS exhibited a different chemical profile, with the predominance of monoterpene hydrocarbons (66.1%), such as myrcene (24.3%), terpinolene (10.6%), and α-pinene (11.4%). Here, the sesquiterpenes were in lower amounts (26.4%) and were represented by (*E*)-caryophyllene (19.3%) and α-humulene (6.4%). It is important to note that the cannabinoid fraction was almost absent, with cannabidiol at trace levels or missing in the samples analyzed.

### 2.2. Toxicity of C. sativa Essential Oils on Mosquito Larvae and Pupae

**Figure 2:**
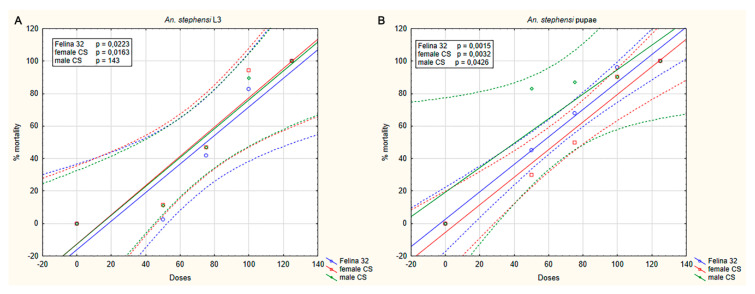
Dose–response curve of insecticidal activity of *C. sativa* essential oils (EOs) on *An. stephensi* preadults. Larvae (**A**) and pupae (**B**) were exposed for 24 h to different dilutions of hemp EOs (50, 75, 100, and 125 ppm). The level of susceptibility of both larvae and pupae to the EOs was found to be statistically significant (as reported in figure). EO samples, Felina 32 inflorescence; CS female inflorescence; CS male inflorescence.

**Figure 3:**
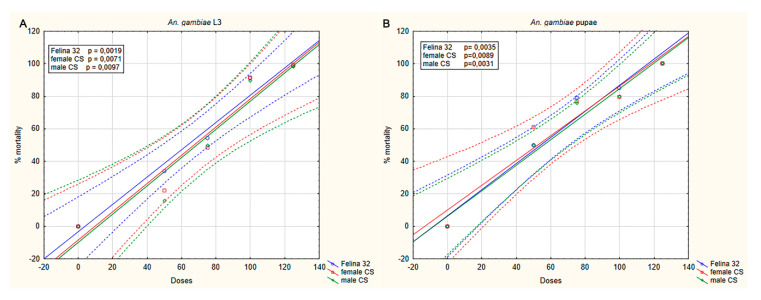
Dose–response curve of insecticidal activity of *C. sativa* essential oils (EOs) on *An. gambiae* preadults. Larvae (**A**) and pupae (**B**) were exposed for 24 h to different dilutions of EOs (50, 75, 100, and 125 ppm). The level of susceptibility of both larvae and pupae to the EOs was found to be statistically significant (as reported in figure). EO samples, Felina 32 inflorescence; CS female inflorescence; CS male inflorescence.

*An. stephensi* and *An. gambaie* larvae and pupae were exposed to different dilutions of the three hemp EOs obtained from the monoecious Felina 32 and female and male inflorescences of CS, and the mortality was recorded after 24 h of treatment. The dose–response curves showed an insecticidal effect of the three EOs on larvae and pupae of *An. stephensi* (Figure 2) and *An. gambiae* (Figure 3). No effects were revealed when the mosquitoes were exposed to pure breeding in water or DMSO (control groups).

A dose–response effect was observed in the insecticidal activity of the three EOs in both *An. stephensi* and *An. gambiae*. In *An. stephensi*, mortality caused by hemp EO at 100 ppm was 82.7% and 100% (Felina 32), 90.2% and 94.2% (CS female), and 89.8% and 90.5% (CS male) on larvae and pupae, respectively. At the same concentration (100 ppm), the mortality produced on *An. gambiae* was 91.1% and 84.9% (Felina 32), 91.6% and 79.6% (CS female), and 89.8% and 79.7% (CS male) on larvae and pupae, respectively. Mortality of 100% was observed when the mosquitoes (L3 and pupae) were exposed at the maximus dilution of the three EOs.

LC_50_ values were in the range 73.50 to 78.80 ppm for larvae and 20.13 to 67.19 ppm for pupae, in *An. stephensi* and *An. gambiae*, respectively. Results for each EO showed a lower susceptibility of larvae compared to pupae (Table 2).

### 2.3. Effect of Hemp Essential Oils in HaCaT and NHF A12 Cell Lines

**Figure 4:**
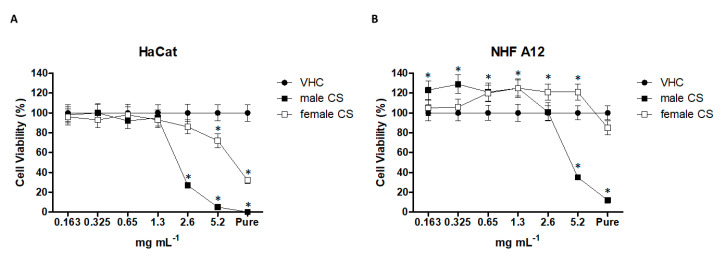
(**A**) HaCaT and (**B**) NHF A12 viability after treatment with different concentrations of EOs. Both cell lines were treated with different concentrations of male CS and female CS EOs, and cell viability was evaluated after 24 h post-treatment. * *p <* 0.05 vs vehicle.

The cytotoxic effect of hemp EOs (derived from male and female inflorescences of cv CS) was evaluated in HaCaT (human keratinocytes) and NHF A12 fibroblasts cell lines by MTT assay (Figure 4). Cells were treated with different dilutions, from pure (1040 mg mL^−1^) to 0.163 mg mL^−1^, for 24 h. EO from male CS inflorescence showed higher cytotoxicity than EO from female CS. However, NHF A12 were more resistant (Figure 4). The IC_50_ values for the EO from male CS inflorescence were 2.23 ± 0.09 and 3.71 ± 0.1 mg mL^−1^, on HaCaT and NHF A12 cells, respectively, while for the EO from female CS inflorescence, the IC_50_ values were higher than 5.2 mg mL^−1^. To further evaluate the potential anti-inflammatory effects of hemp EOs, we firstly tested the ones obtained from male and female inflorescences of cv CS in both cell lines, at 0.65 mg mL^−1^.

#### 2.3.1. Essential Oil Effects on Cell Damage.

**Figure 5:**
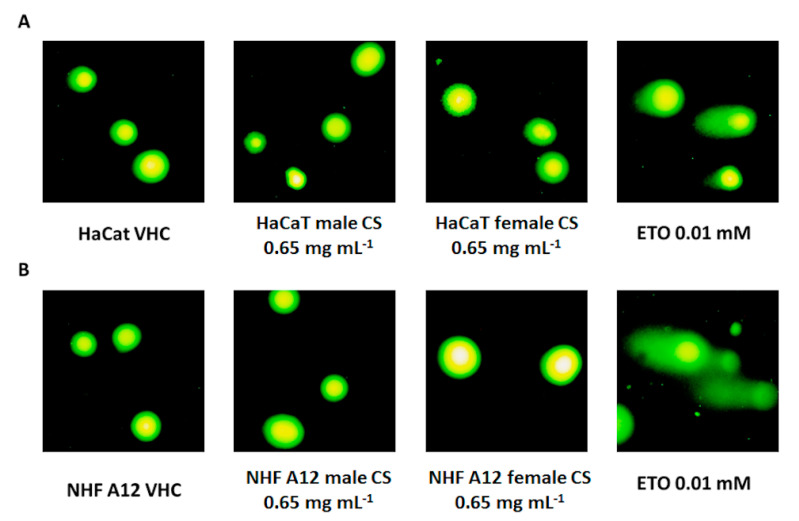
Comet assay. (**A**) HaCaT and (**B**) NHF A12 cell lines were treated with EOs from female and male inflorescences of cv CS at 0.65 mg mL^−1^ and cell damage was analyzed by Comet assay, 24 h post-treatment. ETO was used as a positive control.

In order to evaluate the potential cytotoxic effects, the EOs from male and female inflorescences of cv CS were tested on HaCaT and NHF A12 cell lines. The results showed that the two EOs did not display cellular damage and DNA fragmentation at 0.65 mg mL^−1^. Instead, ETO, used at a cytotoxic dose, evidenced cell damage and DNA fragmentation. Therefore, the EOs at the appropriate dilution did not cause any damage to either cell line (Figure 5).

#### 2.3.2. Hemp Essential Oils Reduced Proinflammatory Gene Expression in ETO-treated HaCaT and NHF A12 Cell Lines.

**Figure 6:**
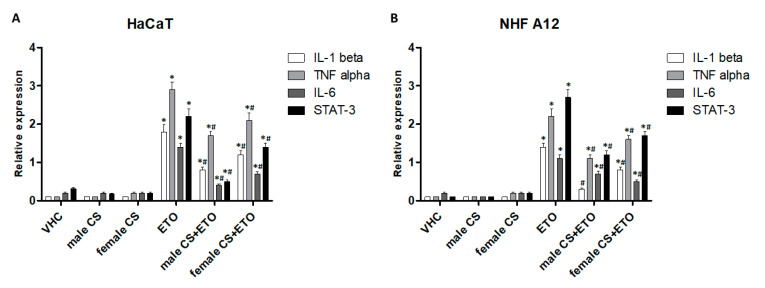
Transcriptional modulation of cytokines genes in HaCaT (**A**) and NHF A12 (**B**) cell lines. Cell lines treated with hemp EOs from female and male inflorescences of cv CS (0.65 mg mL^−1^) alone or in combination with ETO (0.01 mM). Gene expression was represented as relative expression compared to GAPDH. * *p* < 0.05 vs vehicle, # *p* < 0.05 vs ETO.

Through the analysis of cytokine gene expression profiles, we evaluated the modulation of the main proinflammatory genes in HaCaT and NHF A12 cell lines treated with the two EOs from male inflorescences of cv CS and their combination with ETO, at 24 h post-treatment (Figure 6). ETO, at low doses, stimulated an inflammatory effect. The results showed that the EOs did not modulate the basal levels of the cytokines analyzed and the transcription factor STAT-3. However, both EOs were effective in reducing the ETO-induced inflammatory pathways.

#### 2.3.3. Hemp Essential Oils Reduced Proinflammatory Protein Release in ETO-treated HACAT and NHF A12 Cell Lines

**Figure 7:**
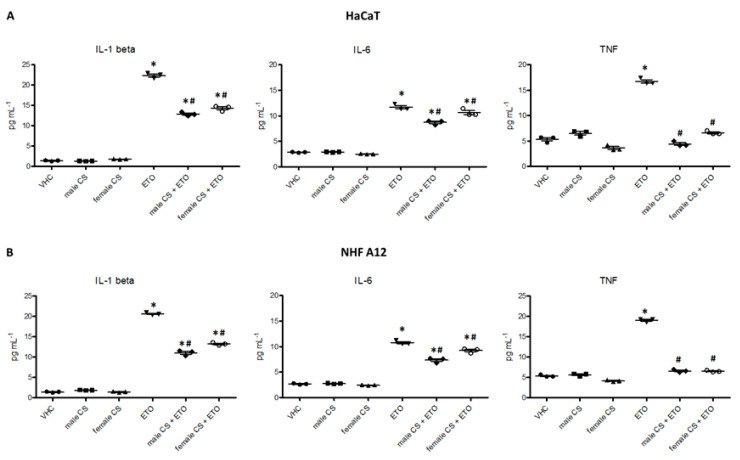
Hemp EOs reduced Etoposide inflammatory effect in HaCaT (**A**) and NHF A12 (**B**) cell lines. The IL-1 beta, IL-6, and TNF protein levels were quantified in culture medium. Amounts of cytokines were reported as pg/mL. * *p* < 0.05 vs vehicle, # *p* < 0.05 vs ETO.

Cytokine levels in culture medium was evaluated in HaCaT and NHF A12 cell lines treated with the two EOs from male and female inflorescences of cv CS and their combination with ETO, at 24 h post-treatment (Figure 7).

The results showed that the two hemp EOs did not modulate the basal levels of the cytokines analyzed, but both were effective in reducing the ETO-induced inflammatory cytokines released in the culture medium.

### 2.4. Preparation and Characterization of C. sativa Essential Oil Nanoemulsions

NEs are colloidal systems that offer the great advantage of being able to encapsulate a higher amount of oil phase compared to similar nanosystems, i.e., microemulsions. Moreover, they require a very low amount of surfactant with a SOR (surfactant–oil ratio) recorded between 1 and 2, compared to that of microemulsions, which is generally higher than 2 (SOR > 2). However, NEs are energetically disadvantaged nanosystems; thus, an external energetic input is required for their formation. In this respect, for the achievement of EO NEs, samples were subjected to a pressure of 130 MPa, selected after the evaluation of different operating pressure, from 50 to 150 MPa.

After a preliminary screening using a model oil phase, the quantitative composition of the NEs has been selected as follows: 6% (*w*/*w*) of the oil phase was emulsified in the aqueous medium through the addition of 4% (*w*/*w*) of surfactant, Tween 80. However, this composition was not suitable for the encapsulation of the EO from female inflorescences of cv CS; in fact, formulations A8 and A9 recorded a high PDI (polydispersity index) value at t0, indicating the polydispersity of the size distribution, with a particle population with a mean diameter higher than 500 nm. Moreover, after 1 month of storage, instability phenomena, i.e., creaming and phase separation, occurred in such samples (Table 3).

In this respect, the EO percentage was decreased to 4% (*w*/*w*) in order to obtain a stable monodispersed system, having a mean diameter within 200 nm and a PDI value lower than 0.3. Although the reduction of the oil phase led to slight system improvement, samples A8B and A9B revealed their instability between 1 and 2 months after their preparation, undergoing phase separation (Table 3).

Then, we decided to add ethyl oleate, commonly used as an oily phase in such systems, to overcome instability issues related to the physicochemical properties of the EO. In fact, ethyl oleate showed good solvent properties, making the oil phase more homogeneous by allowing a better dispersion and encapsulation of active ingredients. The minimum EO–ethyl oleate ratio allowing a good dispersion of the EO in the oil phase was fixed at 1:1. Thus, keeping the amount of oil phase constant at 6% (*w*/*w*), systems were composed of 3% EO, 3% ethyl oleate, 4% Tween 80, and water (Table 3).

Samples A10 and A21 showed a monomodal size distribution with a size in the nanometric range, below 200 nm, which is the limit established by some authors for the definition of a nanosystem.

### 2.5. Stability of C. sativa Essential Oil Nanoemulsions

**Figure 8:**
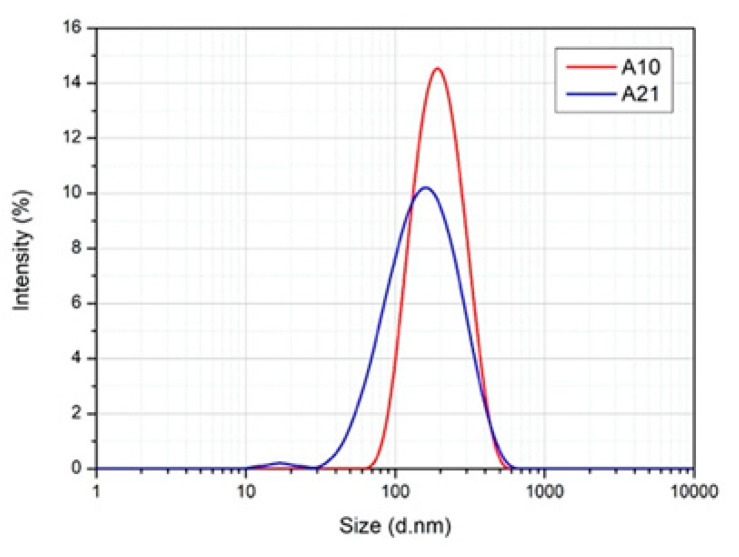
DLS traces of A10 and A21 after a storage period of six months.

Sample A10 and A21 showed optimal stability at room temperature, evaluated at different time points, for a total storage period of six months. As reported in Figure 8, the size of the oil droplets remained almost unchanged, confirming the thermodynamic stability of these systems. In particular, S10 has Z-average and PDI values of 177.9 nm and 0.173, respectively, while S21 has Z-average and PDI values of of 138.4 nm and 0.245, respectively.

## 3. Discussion

The hemp EO obtained from the female inflorescence of cv CS had a higher yield than that of male inflorescences, and this was in agreement with the results reported by Nagy et al. [28].

The three EOs obtained from monoecious, female, and male inflorescences of hemp cv Felina 32 and CS exhibited different chemical profiles (Figure 1).

As for Felina 32 EO, a previous work by Fiorini et al. [1] confirmed that sesquiterpene hydrocarbons, followed by monoterpene hydrocarbons, were the main volatile groups characterizing this variety. Consistently, the EO obtained from the fresh inflorescences of cv Felina 32, which was characterized by Tabari et al. [20], showed (*E*)-caryophyllene and α-pinene as the predominant components.

In our EO samples, cannabidiol was detected in poor amounts in Felina 32 and CS female dry inflorescences (0.1 and 0.2%, respectively). In many other works, such as that by Novak et al. [29], relatively consistent rates of cannabidiol (9.8–10.9%) were found in *C. sativa* EOs. Other differences were noticed in the balance between the two main hemp volatile fractions, namely, monoterpenes and sesquiterpenes, with the former being dominant in the EO chemical profiles obtained from female inflorescences of cv CS, and the latter representing the main fraction of the ones obtained from monoecious Felina 32 and CS male inflorescences.

Based on these results, we conclude that the type of cultivar (e.g., monoecious vs. dioecious), the status of the plant material (e.g., fresh vs. dry) and extraction technique (e.g., steam distillation vs. hydrodistillation) are key factors influencing the chemical profile of hemp EO. On this basis, we evaluated the biological potential, namely mosquitocidal, toxicological, and immunomodulatory effects of these EOs.

Innovative methods to control the mosquito vector populations are strongly required [30]. Larvae and pupae represent attractive targets for pesticides because these developmental stages are aquatic and breed in water. The employment of chemical compounds in the water sources (including drinking water), however, causes many risks to people and the environment, whereas natural and eco-friendly pesticides, especially those obtained from plants, are extremely promising [31,32]. Insecticides of plant origin showed several features including biodegradability, nontoxicity for non-target organisms, and cost-efficacy [33,34]. The EOs extracted from different plants can contain several molecules with larvicidal activity against mosquitoes [16,35,36], and many compounds are currently well-known [37,38]. The requirement for the identification of novel and effective plant products represents a crucial aspect with the purpose of improving insecticide formulations in order to replace chemical insecticides to control mosquitoes [33].

The EOs from hemp cultivars have been studied as potential biopesticides in crop protection or for the control of mosquito and fly vectors [39]. In previous studies, hemp EOs have shown toxicity against mosquitoes and snails [2,3,40,41]. Our studies demonstrated the strong toxicity of three EOs of hemp (CS and Felina varieties) against larvae and pupae of the main malaria vectors *An. stephensi* and *An. gambiae*, in Asia and Africa, respectively. The lower LC_50_ of pupae compared to larvae could be due to the different metabolic traits of these developmental stages of the mosquitoes. Our results demonstrated that the analyzed hemp EOs are highly active against both preadult stages, with LC_50_ values lower than 80 ppm. This value represents an important threshold to consider a natural product as a valuable ingredient for the formulation of botanical insecticides [42]. The LC_50_ values found in this study are lower than the average of those of many EOs extracted from plants [43], with the advantage of using an industrial waste product.

The mortality of mosquitoes may be caused mainly by α-pinene, myrcene, terpinolene, (*E*)-caryophyllene, and α-humulene, which occurred in significant amounts in the hemp EOs analyzed. In fact, myrcene and α-pinene were among the most abundant constituents of *C. sativa* EO studied by Bedini et al. [41], which were revealed to be effective against the Asian tiger mosquito *Aedes albopictus*. In the publications by Benelli et al. [2,3], the toxicity of hemp EOs on *Culex quinquefasciatus* was linked to the presence of α-pinene, myrcene, (*E*)-caryophyllene, and terpinolene as the major compounds. (*E*)-Caryophyllene and α-humulene, the main sesquiterpenes of hemp EO, have shown notable mosquitocidal and acaricidal properties [29,44].

To our knowledge, information on the anti-inflammatory effect of hemp EOs is very limited. On the other hand, some authors [45] reported that CBD-enriched extracts can reduce IL-8 and Nf-κB pathways. However, in this study, we demonstrated that the CBD-free hemp EOs did not induce an inflammatory condition, but, additionally, were able to revert an inflammatory condition, reducing the release of the cytokine induced by ETO, on skin cell lines, suggesting that they could be safely used by operators. Among the various hemp EO constituents detected, (*E*)-caryophyllene and α-humulene have been reported to exert anti-inflammatory effects in vivo through a reduction of IL-1β and TNF-α release. This effect was comparable to that of the positive control drug, dexamethasone [46].

Despite hemp EOs showed potential as ingredients of botanical insecticides, their widespread use should be restricted due to the poor physicochemical properties of EOs in general. In fact, they show several limitations, i.e., high volatility, thermal decomposition, low water solubility, and stability issues [47]. In this respect, nanotechnology could support the exploitation of EOs through their encapsulation into stable formulations, i.e., NEs, overcoming such limitations [48]. Moreover, being biphasic colloidal systems, NEs allow the dispersity of the EOs into an aqueous medium, a very important property for a pesticide. Thus, hemp EOs based NEs were prepared. At the end of screening on the quali/quantitive composition of the system, 3% of hemp EO was encapsulated into a nanoemulsified system to assure its stability over time. Although several studies reported an increased bioavailability of EOs encapsulated into nanoformulations, further investigations about the insecticidal effectiveness of hemp EO-based NEs are required.

## 4. Materials and Methods

### 4.1. Plant Material

The monoecious inflorescences of *Cannabis sativa* cv Felina 32 (Assocanapa, National Coordination for Canapiculture, Italy, http://www.assocanapa.org/chi.htm) were obtained from a cultivation sited in Fiuminata (central Italy, 43°11′11′′ N, 12°56′24′′ E, 318 m a.s.l.) and collected at the end of July 2017. Female and male inflorescences of *C. sativa* cv CS (Carmagnola Selezionata, Assocanapa, Italy), were harvested at first and second half of September 2018 in another cultivation of Fiuminata (central Italy, 43°10′40′′ N, 12°56′59′′ E, 451 m a.s.l.). Herbarium specimens of the two cultivars were authenticated by Prof. F. Maggi and deposited at the Herbarium of the Centro Ricerche Florisitche dell’Appennino (APP), Barisciano, L’Aquila, Italy, under the codes APP 57789 and APP 60530. In both cases, the plant material consisted of 15–20 cm composite samples, including flowers, leaflets, and upper stems.

### 4.2. Reagents

Etoposide (ETO) was purchased from Abcam (Milan, Italy) and diluted in sterilized water at a concentration of 50 mM. Hemp EOs used are those from monoecious inflorescence of Felina 32 and from male and female inflorescences of CS. Each EO was used pure or diluted in DMSO for the analysis.

### 4.3. Mosquitoes

*An. gambiae* (G3 strain) and *An. stephensi* (Liston strain) were reared in the insectary at 29 °C ± 2 and 85% ± 5 relative humidity, with photoperiod (12:12 light:dark). Adult insects were maintained with 5% sucrose solution ad libitum, and adult females were fed on mouse blood for egg-laying. Eggs were collected on wet filter paper for 48 h before hatching. Larvae were maintained in spring water and fed daily with commercial fish food.

### 4.4. Cell Lines

An immortalized human keratinocyte (HaCaT) cell line (Creative Bioarray, Shirley, NY, USA) was cultured in DMEM supplemented with 10% fetal bovine serum (FBS), 2 mM L-glutamine, 100 IU mL^−1^ of penicillin, 100 µg of streptomycin (Lonza, Allendale, NJ, USA), 1 mM sodium pyruvate (Lonza) and maintained at 37 °C with 5% CO_2_ and 95% humidity. Primary human stabilized NHF A12 fibroblast cell line (ATCC, LGC Standards, Milan, Italy) was cultured in DMEM supplemented with 10% fetal bovine serum (FBS), 2 mM L-glutamine, 100 IU mL^−1^ of penicillin, 100 µg of streptomycin (Lonza), 1 mM sodium pyruvate (Lonza) and maintained at 37 °C with 5% CO_2_ and 95% humidity.

### 4.5. Steam Distillation

After being cut into small parts, 2500 g of fresh male inflorescences of cv CS were subjected to steam distillation (SD), by an Albrigi Luigi E0106 (Stallavena di Grezzana-Verona, Italy) stainless steel apparatus (capacity 20 L) for 4 h, with 2 L of distilled water at the bottom of it, to produce the steam. This device was equipped with a steel Clevenger-type apparatus and a glass burette for the collection of the EO. Once separated from the aqueous layer, the EO was collected in dark vials sealed with PTFE-silicon septa, and stored at 4 °C until analysis. The EO yield was calculated on a dry weight basis.

### 4.6. Hydrodistillation

Hydrodistillation (HD) was performed for dried samples of cv Felina 32 monoecious inflorescences and cv CS female inflorescences. In both cases, we used 2000 g of plant material soaked in a 20 L glass flask filled with 11 L of water that was heated by a mantle system Falc MA (Falc Instruments, Treviglio, Italy) for 4 h. The EOs were recovered by a glass Clevenger-type apparatus. In this case, the obtained EOs were kept in the dark glass vials in the fridge before chemical characterization and biological assays. The EO yield was calculated on a dry weight basis.

### 4.7. GC-MS Analysis

The chemical composition of hemp EOs was analyzed through an Agilent 6890N GC-MS system coupled to a 5973N single quadrupole detector mass spectrometer. Separation was provided by a HP-5MS capillary column (5% phenylmethylpolysiloxane, 30 mL × 0.25 mm i.d., 0.1 µm f.t., Agilent, Santa Clara, CA, USA). The temperature program was as follows: 60 °C for 5 min, then 4 °C min^−1^ up to 220 °C, finally 11 °C min^−1^ to 280 °C, maintained for 15 min, for a total run time of 65 min. The temperature of the injector and detector was 280 °C. Helium (He) was the carrier gas, with a flow rate of 1 mL min^−1^ and a 1:50 split ratio. The chromatograms were acquired in full scan in the range 29.0–400.0 uma, using electron-impact (EI, 70 eV) mode. Dilution 1:100 of essential oils in *n*-hexane was injected (2 µL) into the GC-MS system. The MSD ChemStation (Agilent, Version G1701DA D.01.00) and the NIST Mass Spectral Search Program were employed for the data analysis. The identification of the principal compounds was achieved by the correspondence of retention indices and mass spectra to those of ADAMS, NIST 17, FFNSC2, and WILEY 275 libraries. Furthermore, the analytical standards available in the laboratory (Sigma-Aldrich, Milan, Italy) were used for further confirmation. The relative peak area percentages were obtained by area normalization without using correction factors [49].

### 4.8. Insecticidal Assays

The larvicidal effect of the EOs was evaluated according to standard procedures established by the WHO [50]. Three different hemp EOs were analyzed: (i) Felina 32 dioecious inflorescence, (ii) CS female inflorescence, (iii) CS male inflorescence. For the treatment of mosquitoes, the EOs were dissolved in dimethyl sulfoxide (DMSO) 1:10. Twenty-five larvae L3 (third instar stage) and twenty-five pupae were placed in plastic cups containing water. Treatment was carried out using different dilutions of the three essential oils preparations: 50, 75, 100, and 125 ppm. Control groups were prepared adding pure breeding water or DMSO (200 µL). Mortality of larvae and pupae was observed after 24 h. Three independent experiments were performed.

### 4.9. MTT Assay

Three thousand cells per well were seeded in 96-well plates. After one day of incubation, hemp EOs (from male and female inflorescence of CS) and vehicles were added, and six replicates were used for each treatment. At the indicated time point, 24 h, cell viability was investigated by adding 0.8 mg mL^−1^ of 3-(4,5-dimethylthiazol-2yl)-2,5 diphenil tethrazolium bromide MTT (Sigma Aldrich) to the media. The reaction was given by mitochondrial reductase which changed MTT (tetrazolium salt) colour from yellow to purple, forming formazan crystal. After 3 h, plates were centrifuged, the supernatant was removed, and the pellet of salt crystals was solubilised with 100 µL well^−1^ of DMSO. The absorbance of the sample against a background control (medium alone) was measured at 570 nm using an ELISA reader microliter plate (BioTek Instruments, Winooski, VT, USA).

### 4.10. Gene Expression Analysis by TaqMan Array

Total RNA was extracted with the RNeasy Mini Kit (Qiagen), and cDNA was synthesized using the High-Capacity cDNA Archive Kit (Applied Biosystems, Foster City, CA, USA) according to the manufacturer’s instructions. Quantitative real-time polymerase chain reactions (qRT-PCR) were performed with TaqMan Array (Thermo Fisher) using the iQ5 Multicolor Real-Time PCR Detection System (Bio-Rad, Hercules, CA, USA). The PCR parameters were 10 min at 95 °C followed by 40 cycles at 95 °C for 15 s and 60 °C for 40 s. The relative amount of target mRNA was calculated by the 2^−ΔΔCt^ method, using GAPDH as a housekeeping gene. All samples were assayed in triplicates on the same plate. Measurement of GAPDH levels was used to normalize mRNA contents, and target gene levels were calculated by the 2^−ΔΔCt^ method. The gene list is presented in Figure 6.

### 4.11. ELISA Assay

The concentration of inflammatory cytokines released from HaCaT and NHF A12 cell lines after treatment with ETO combined and not with hemp EOs, was evaluated in cell culture supernatants of cells treated for 24 h. The levels of cytokines were evaluated in duplicated and in two independent experiments using ELISA kit (Abcam). Cytokine concentrations were calculated by plotting the OD values in the equation curve obtained with standard, according to the manufacturer’s protocol.

### 4.12. Alkaline Comet Assay

Cells were plated in six-well plates (3 × 10^4^ cells well^−1^) one day before treatment exposure. Semiconfluent cultures were treated for 24 h with hemp EOs from CS male and female inflorescences (0.65 mg mL^−1^) and 10 µM of ETO. Cells treated with vehicle were included in all series. The Comet assay was performed under alkaline conditions following the ABCAM protocol. Briefly, after exposure to treatments, the cells were resuspended in 1× PBS and added to 75 µL of molten (37 °C) 0.5% low-melting-point agarose gel to achieve a cell concentration of 1 × 10^5^ cells mL^−1^. The agarose was pipetted onto the Comet slides. Slides were stored in the dark at 4 °C for 10 min before adding prechilled lysis buffer for 45 min at 4 °C in the dark. The slides were immersed in freshly prepared alkaline solution (0.25 M NaOH containing 0.1 µM EDTA, pH 12.6) for 30 min at the same conditions. Slides were then removed and washed twice with TBE buffer for 5 min. Gel electrophoresis was performed at 1 V cm^−1^ for 20 min (running amperage 3–5 mA with the distance between the two electrodes of 25 cm). The Comet slides were washed with 70% ethanol for 5 min and air-dried for 1 h at room temperature. A total of 100 µL of diluted SYBR Green solution was placed onto each dried agarose circle. The slides were then read with a fluorescence microscope (LEIKA).

### 4.13. Statistical Analysis

LC_50_ values were obtained by nonlinear regression using GraphPad Prism version 5.0 (GraphPad Software, San Diego, CA, USA). The graphs related to the mortality effect of the EOs on larvae and pupae of *An. stephensi* and *An. gambiae*. Data were plotted using STATISTICA software version 6.0. The correlation between the mortality and the EOs doses was assessed using linear regression analysis (Fisher–Snedecor test) using GraphPad Prism version 8.0 (GraphPad Software, San Diego, CA, USA). For MTT, RT-PCR, and Elisa assay, the data presented represent the mean and standard deviation of at least two independent experiments. The statistical significance was determined by Student’s *t*-test and by one-way ANOVA; *, # *p* < 0.01 or *p* < 0.05 (as described in the figure’s legends). The statistical analysis was performed using Prism 5.0a (Graph Pad).

### 4.14. Preparation of EO-Based Nanoemulsions

NEs were obtained through a high-energy method, by using a high-pressure homogenizer. Briefly, different percentages (6%, 4% *w*/*w*) of CS female inflorescence EO or a 6% (*w*/*w*) of a binary mixture of EO and ethyl oleate (1:1 ratio) were added dropwise to a 4% (*w*/*w*^−1^) of surfactant (Tween 80) aqueous solution under high-speed stirring (Ultraturrax T25 basic, IKA^®^ Werke GmbH & Co.KG, Staufen, Germany) for 5 min at 9500 rpm. The obtained emulsions were then subjected to the homogenization process by means of French Pressure Cell Press (American Instrument Company, AMINCO, MY, USA) for four cycles at the pressure of 130 MPa.

### 4.15. Nanoemulsions Characterization

Visual inspection of formulations was done by a polarizing optical microscope (MT9000, Meiji Techno Co Ltd., Chikumazawa, Miyoshi machi, Iruma-gun, Saitama 354-0043, Japan) equipped with a 3 megapixel CMOS camera (Invenio 3S, DeltaPix, Denmark). Particle size measurements were performed through dynamic light scattering (DLS) technique. DLS analyses were carried out using a Zetasizer nanoS (Malvern Instruments, Worcestershire, UK) equipped with backscattered light detector working at 173°. Samples (1 mL) were inserted into disposable cuvettes and analyzed at 25 °C, following a temperature equilibration time (180 s). The analysis was performed at different time points: 0 day (t0), 1 month (t1), 3 months (t3), and 6 months (t6).

## 5. Conclusions

In the present work, we investigated the potential application of the EOs obtained from industrial hemp as mosquitocidal agents. The three EOs tested, coming from different cultivars and plant parts, showed efficacy in killing the two malaria vectors, *An. gambiae* and *An. stephensi*. The LC_50_ values found in this study are, on average, lower than those of many plant EOs, with the advantage of using an industrial waste product. Notably, this work highlighted that the male inflorescence, which is usually discarded during the industrial hemp processing, may be a sustainable source of larvicidal compounds. The bioactivity observed in the larvicidal assays may be entirely attributed to the terpenoid fraction of the EOs since the cannabinoid one was almost absent. Moreover, the toxicological assays and Comet assays put in evidence the safety of the hemp EOs at certain doses on human cell lines, whereas the gene expression analysis, together with the ELISA, showed, for the first time, their anti-inflammatory potential and safety profile. This result may open new avenues of use for hemp EOs in pharmaceuticals and cosmeceuticals. For this reason, stable nanoemulsions were developed to guarantee better water dispersity and chemical stability over time, while reducing the high volatility of EOs, making them ready-to-use products to be further investigated as larvicidal and anti-inflammatory agents.

## Figures and Tables

**Table 1:** Chemical characterization of the hemp essential oils obtained from monoecious, male, and female inflorescences of hemp, cv Felina 32 and Carmagnola Selezionata (CS).

N	Component ^a^	RI Calc. ^b^	RI Lit. ^c^	% Felina 32	Carmagnola CS	
					% Female	% Male
1	Tricyclene	914	921	0.2		
2	α-Thujene	921	924	0.1	0.1	
3	α-Pinene	926	932	15.1	11.4	8.0
4	Camphene	939	946	0.2	0.2	
5	Sabinene	966	967		0.2	
6	β-Pinene	968	974	5.0	5.7	3.1
7	Myrcene	989	988	11.8	24.3	10.6
8	α-Phellandrene	1003	1002		0.4	
9	δ-3-Carene	1008	1008		0.4	
10	α-Terpinene	1014	1014	0.1	0.3	
11	Limonene	1025	1024	6.0	6.7	4.1
12	(1,8)-Cineole	1027	1026	3.5	1.4	1.6
13	(*Z*)-β-Ocimene	1037	1032	0.1	0.2	
14	(*E*)-β-Ocimene	1047	1044	0.8	2.3	0.3
15	γ-Terpinene	1056	1054	0.3	0.4	0.2
16	*cis*-Sabinene Hydrate	1064	1065	0.2		
17	Terpinolene	1085	1086	0.9	13.5	0.3
18	Linalool	1101	1095		0.8	
19	(*Z*)-Caryophyllene	1407	1408	0.3		0.6
20	(*E*)-Caryophyllene	1409	1417	34.8	19.3	47.2
21	α-*trans*-Bergamotene	1430	1432	1.0		0.5
22	α-Humulene	1443	1452	11.4	6.4	15.1
23	*allo*-Aromadendrene	1450	1458	0.6		0.8
24	(*E*)-β-Farnesene	1456	1454	0.9		
25	β-Selinene	1476	1519	1.5	0.4	1.7
26	α-Selinene	1485	1498	1.0	0.3	1.2
27	δ-Cadinene	1519	1522	0.1		
28	Selina-3,7(11)-diene	1531	1545	0.5		0.6
29	Caryophyllene oxide	1571	1582	2.1	2.2	3.6
30	Humulene epoxide II	1593	1608	0.4	0.5	0.5
31	Cannabidiol	2419	2430	0.1	0.2	
	Total identified (%)			99.0	97.6	100
	monoterpene hydrocarbons			40.6	66.1	26.6
	oxygenated monoterpenes			3.7	2.2	1.6
	sesquiterpene hydrocarbons			52.1	26.4	67.7
	oxygenated sesquiterpenes			2.5	2.7	4.1
	cannabinoids			0.1	0.2	

^a^ Components were eluted from a HP-5MS (30 mL × 0.25 mm i.d., 0.1 mm f.t.) column. ^b^ Liner retention index, experimentally determined using a mixture C8-C30 of alkanes. ^c^ Retention index value taken from literature.

**Table 2:** LC_50_ values of essential oils from monoecious, male, and female inflorescences of hemp, cv Felina 32 and Carmagnola CS against larvae and pupae of *An. stephensi* and *An. gambiae*.

Mosquito Species	LC_50_^a^					
	Larvae			Pupae		
	Felina 32 ^b^	Female CS ^b^	Male CS ^b^	Felina 32 ^b^	Female CS ^b^	Male CS ^b^
*An. stephensi*	78.8	75.12	75.23	54.41	67.19	20.13
*An. gambiae*	73.5	75.54	75.04	50.06	41.51	50.27

^a^ LC_50_ is expressed in ppm. ^b^ EO samples, Felina 32 inflorescence; CS female inflorescence; CS male inflorescence.

**Table 3:** Composition and physicochemical properties of hemp EO-based nanoemulsions.

Sample	Essential Oil	Surfactant	Other Components	Description
A8	*C. sativa* cv CS 6% (male inflorescences)	Tween 80 4%		-Polydispersed system at t0 -Instability phenomena after 1 month
A9	*C. sativa* cv CS 6% (female inflorescences)	Tween 80 4%		-Polydispersed system at t0 -Instability phenomena after 1 month
A8B	*C. sativa* cv CS 4% (male inflorescences)	Tween 80 4%		-Polydispersed system at t0 -Instability phenomena between 1 and 2 months
A9B	*C. sativa* cv CS 4% (female inflorescences)	Tween 80 4%		-Polydispersed system at t0 -Instability phenomena between 1 and 2 months
A10	*C. sativa* cv CS 3% (female inflorescences)	Tween 80 4%	Ethyl oleate 3%	-Mean diameter ≈ 200 nm PDI < 0.2 -Stable system after 6 months
A21	*C. sativa* cv CS 3% (male inflorescences)	Tween 80 4%	Ethyl oleate 3%	-Mean diameter ≈ 200 nm PDI < 0.2 -Stable system after 6 months

## References

[1] Fiorini D., Molle A., Nabissi M., Santini G., Benelli G., Maggi F. (2019). Valorizing industrial hemp (*Cannabis sativa* L.) by-products: Cannabidiol enrichment in the inflorescence essential oil optimizing sample pre-treatment prior to distillation. Ind. Crops Prod..

[2] Benelli G., Pavela R., Lupidi G., Nabissi M., Petrelli R., Kamte S.L.N., Cappellacci L., Fiorini D., Sut S., Dall’Acqua S. (2018). The crop-residue of fiber hemp cv. Futura 75: From a waste product to a source of botanical insecticides. Env. Sci. Pollut. Res..

[3] Benelli G., Pavela R., Petrelli R., Cappellacci L., Santini G., Fiorini D., Sut S., Dall’Acqua S., Canale A., Maggi F. (2018). The essential oil from industrial hemp (*Cannabis sativa* L.) by-products as an effective tool for insect pest management in organic crops. Ind. Crops Prod..

[4] Petrelli R., Ranjbarian F., Dall’Acqua S., Papa F., Iannarelli R., Kamte S.L.N., Vittori S., Benelli G., Maggi F., Hofer A. (2017). An overlooked horticultural crop, *Smyrnium olusatrum*, as a potential source of compounds effective against African trypanosomiasis. Parasitol. Int..

[5] Kamte S.L.N., Ranjbarian F., Cianfaglione K., Sut S., Dall’Acqua S., Bruno M., Afshar F.H., Iannarelli R., Benelli G., Cappellacci L. (2018). Identification of highly effective antitrypanosomal compounds in essential oils from the Apiaceae family. Ecotoxicol. Environ. Saf..

[6] Benelli G., Pavela R., Iannarelli R., Petrelli R., Cappellacci L., Cianfaglione K., Afshar F.H., Nicoletti M., Canale A., Maggi F. (2017). Synergized mixtures of Apiaceae essential oils and related plant-borne compounds: Larvicidal effectiveness on the filariasis vector *Culex quinquefasciatus* Say. Ind. Crops Prod..

[7] Vitali L.A., Beghelli D., Nya P.C.B., Bistoni O., Cappellacci L., Damiano S., Lupidi G., Maggi F., Orsomando G., Papa F. (2016). Diverse biological effects of the essential oil from Iranian *Trachyspermum ammi*. Arab. J. Chem..

[8] Woguem V., Fogang H.P., Maggi F., Tapondjou L.A., Womeni H.M., Quassinti L., Bramucci M., Vitali L.A., Petrelli D., Lupidi G. (2014). Volatile oil from striped African pepper (*Xylopia parviflora*, Annonaceae) possesses notable chemopreventive, anti-inflammatory and antimicrobial potential. Food Chem..

[9] World Health Organization (2018). World Malaria Report 2018.

[10] Miller L.H., Baruch D.I., Marsh K., Doumbo O.K. (2002). The pathogenic basis of malaria. Nature.

[11] Hanafi-Bojd A.A., Vatandoost H., Oshaghi M.A., Haghdoost A.A., Shahi M., Sedaghat M.M., Abedi F., Yeryan M., Pakari A. (2012). Entomological and epidemiological attributes for malaria transmission and implementation of vector control in southern Iran. Acta Trop..

[12] Manimaran A., Cruz M.M.J.J., Muthu C., Vincent S., Ignacimuthu S. (2012). Larvicidal and knockdown effects of some essential oils against *Culex quinquefasciatus* Say, *Aedes aegypti* (L.) and *Anopheles stephensi* (Liston). Adv. Biosci. Biotechnol..

[13] Chandre F., Darriet F., Manguin S., Brengues C., Carnevale P., Guillet P. (1999). Pyrethroid cross resistance spectrum among populations of *Anopheles gambiae* s.s. from Cote d’Ivoire. J. Am. Mosq. Contr..

[14] Sedaghat M.M., Dehkordi A.S., Khanavi M., Abai M.R., Mohtarami F., Vatandoost H. (2011). Chemical composition and larvicidal activity of essential oil of Cupressus arizonica E.L. Greene against malaria vector *Anopheles stephensi* Liston (Diptera: Culicidae). Pharmacogn. Res..

[15] Asase A., Oteng-Yeboah A.A., Odamtten G.T., Simmonds M.S.J. (2005). Ethnobotanical Study of Some Ghanaian Anti-Malarial Plants. J. Ethnopharmacol..

[16] Pavela R., Maggi F., Iannarelli R., Benelli G. (2019). Plant extracts for developing mosquito larvicides: From laboratory to the field, with insights on the modes of action. Acta Trop..

[17] Wongsrichanalai C., Pickard A.L., Wernsdorfer W.H., Meshnick S.R. (2002). Epidemiology of drug-resistant malaria. Lancet. Infect. Dis..

[18] Potter D.J. (2009). The Propagation, Characterisation and Optimisation of *Cannabis sativa* L. as a Phytopharmaceutical. Ph.D. Thesis.

[19] Fiorini D., Scortichini S., Bonacucina G., Greco N.G., Mazzara E., Petrelli R., Torresi J., Maggi F., Cespi M. (2020). Cannabidiol-enriched hemp essential oil obtained by an optimized microwave-assisted extraction using a central composite design. Ind. Crops Prod..

[20] Tabari M.A., Khodashenas A., Jafari M., Petrelli R., Cappellacci L., Nabissi M., Maggi F., Pavela R., Youssefi M.R. (2020). Acaricidal properties of hemp (*Cannabis sativa* L.) essential oil against *Dermanyssus gallinae* and *Hyalomma dromedarii*. Ind. Crops Prod..

[21] Baroni A., Buommino E., De Gregorio V., Ruocco E., Ruocco V., Wolf R. (2012). Structure and function of the epidermis related to barrier properties. Clin. Dermatol..

[22] Hänel K.H., Cornelissen C., Lüscher B., Baron J.M. (2013). Cytokines and the skin barrier. Int. J. Mol. Sci..

[23] Pastar I., Stojadinovic O., Yin N.C., Ramirez H., Nusbaum A.G., Sawaya A., Patel S.B., Khalid L., Isseroff R.R., Tomic-Canic M. (2014). Epithelialization in wound healing: A comprehensive review. Adv. Wound Care (New Rochelle).

[24] Kawagishi C., Kurosaka K., Watanabe N., Kobayashi Y. (2001). Cytokine production by macrophages in association with phagocytosis of etoposide-treated P388 cells in vitro and in vivo. Biophys. Acta.

[25] Darst M., Al-Hassani M., Li T., Yi Q., Travers J.M., Lewis D.A., Travers J.B. (2004). Augmentation of chemotherapy-induced cytokine production by expression of the platelet-activating factor receptor in a human epithelial carcinoma cell line. J. Immunol..

[26] Turek C., Stintzing F.C. (2013). Stability of essential oils: A review. Compr. Rev. Food Sci. Food Saf..

[27] Bertoli A., Ruffoni B., Pistelli L., Pistelli L. (2010). Analytical methods for the extraction and identification of secondary metabolite production in ‘in vitro’ plant cell cultures. Adv. Exp. Med. Biol..

[28] Nagy D.U., Cianfaglione K., Maggi F., Sut S., Dall’Acqua S. (2019). Chemical characterization of leaves, male and female flowers from spontaneous Cannabis (*Cannabis sativa* L.) growing in Hungary. Chem. Biodivers..

[29] Novak J., Franz C. (2003). Composition of the essential oils and extracts of two populations of *Cannabis sativa* L. ssp. spontanea from Austria. J. Essent. Oil Res..

[30] Kendall D.A., Yudowski G.A. (2017). Cannabinoid receptors in the central nervous system: Their signaling and roles in disease. Front. Cell. Neurosci..

[31] Subramaniam J., Kovendan K., Mahesh Kumar P., Murugan K., Walton W. (2012). Mosquito larvicidal activity of Aloe vera (Family: Liliaceae) leaf extract and Bacillus sphaericus, against Chikungunya vector, Aedes aegypti. Saudi J. Biol. Sci..

[32] Benelli G., Pavela R. (2018). Beyond mosquitoes—Essential oil toxicity and repellency against bloodsucking insects. Ind. Crops Prod..

[33] Alkofahi A., Rupprecht J.K., Anderson J.E., McLaughlin J.L., Mikolajczak K.L., Scott B.A., Arnason J.T., Philogene B.J.R., Morand P. (1989). Search for new pesticides from higher plants. Insecticides of Plant Origin.

[34] Benelli G., Maggi F., Pavela R., Murugan K., Govindarajan M., Vaseeharan B., Petrelli R., Cappellacci L., Kumar S., Hofer A. (2018). Mosquito control with green nanopesticides: Towards the One Health approach? A review of non-target effects. Environ. Sci. Pollut. Res. Int..

[35] Kovendan K., Murugan K. (2011). Effect of medicinal plants on the mosquito vectors from the different agroclimatic regions of Tamil Nadu, India. Adv. Environ. Biol..

[36] Benelli G., Pavel R., Zorzetto C., Sánchez-Mateo C.C., Santini G., Canale A., Maggi F. (2019). Insecticidal activity of the essential oil from *Schizogyne sericea* (Asteraceae) on four insect pests and two non-target species. Entomol. Gen..

[37] Cheng S.S., Huang C.G., Chen Y.J., Yu J.J., Chen W.J., Chang S.T. (2009). Chemical compositions and larvicidal activities of leaf essential oils from two eucalyptus species. Bioresour. Technol..

[38] Wang J., Zhu F., Zhou X.M., Niu C.Y., Lei C.I. (2006). Repellent and fumigant activity of essential oil from Artemisia vulgaris to *Tribolium eastaneum* (Herbst). J. Stored Prod. Res..

[39] Pavela R., Murugan K., Canale A., Benelli G. (2017). Saponaria officinalis-synthesized silver nanocrystals as effective biopesticides and oviposition inhibitors against *Tetranychus urticae* Koch. Ind. Crops Prod..

[40] Thomas T.G., Sharma S.K., Prakash A., Sharma B.R. (2000). Insecticidal properties of essential oil of *Cannabis sativa* Linn. against mosquito larvae. Entomon.

[41] Bedini S., Flamini G., Cosci F., Ascrizzi R. (2016). Cannabis sativa and Humulus lupulus essential oils as novel control tools against the invasive mosquito *Aedes albopictus* and fresh water snail *Physella acuta*. Ind. Crops Prod..

[42] Pavela R. (2015). Essential oils for the development of eco-friendly mosquito larvicides: A review. Ind. Crops Prod..

[43] Khanavi M., Fallah A., Vatandoost H., Sedaghat M., Abai M.R., Hadjiakhoondi A. (2012). Larvicidal activity of essential oil and methanol extract of Nepeta menthoides against malaria vector *Anopheles stephensi*. Asian Pac. J. Trop. Med..

[44] Da Silva R.C.S., Milet-Pinheiro P., Bezerra da Silva P.C., da Silva A.G., da Silva M.V., Navarro D.M.D.A.F., da Silva N.H. (2015). (*E*)-caryophyllene and α-humulene: *Aedes aegypti* oviposition deterrents elucidated by gas chromatography-electrophysiological assay of *Commiphora leptophloeos* leaf oil. PLoS ONE.

[45] Sangiovanni E., Fumagalli M., Pacchetti B., Piazza S., Magnavacca A., Khalilpour S., Melzi G., Martinelli G., Dell’Agli M. (2019). Cannabis sativa L. extract and cannabidiol inhibit in vitro mediators of skin inflammation and wound injury. Phytother. Res..

[46] Fernandes E.S., Passos G.F., Medeiros R., da Cunha F.M., Ferreira J., Campos M.M., Pianowski L.F., Calixto J.B. (2007). Anti-inflammatory effects of compounds alpha-humulene and (−)-trans-caryophyllene isolated from the essential oil of Cordia verbenacea. Eur. J. Pharmacol..

[47] Pavoni L., Benelli G., Maggi F., Bonacucina G. (2019). Green nanoemulsion interventions for biopesticide formulations. Nano-Biopesticides Today and Future Perspectives.

[48] Pavoni L., Pavela R., Cespi M., Bonacucina G., Maggi F., Zeni V., Canale A., Lucchi A., Bruschi F., Benelli G. (2019). Green Micro-and Nanoemulsions for Managing Parasites, Vectors and Pests. Nanomaterials.

[49] Quassinti L., Bramucci M., Lupidi G., Barboni L., Ricciutelli M., Sagratini G., Papa F., Caprioli G., Petrelli D., Vitali L.A. (2013). In vitro biological activity of essential oils and isolated furanosesquiterpenes from the neglected vegetable *Smyrnium olusatrum* L. (Apiaceae). Food Chem..

[50] World Health Organization (2005). Guidelines for Laboratory and Field Testing of Mosquito Larvicides.

